# Cadaver-based Necrotizing Fasciitis Model for Medical Training

**DOI:** 10.7759/cureus.1168

**Published:** 2017-04-14

**Authors:** Kurt M Mohty, Matthew G Cravens, William J Adamas-Rappaport, Bahareh Amini-Shervin, Steven C Irving, Nicholas Stea, Srikar Adhikari, Richard Amini

**Affiliations:** 1 College of Medicine, University of Arizona; 2 Department of Surgery, University of Arizona; 3 Departamento De Urgencias, Servicio Andaluz de Salud; 4 Department of Emergency Medicine, University of Arizona

**Keywords:** ultrasound, necrotizing fasciitis, education

## Abstract

Necrotizing fasciitis is a devastating infectious disease process that is characterized by extensive soft tissue necrosis along deep fascial planes, systemic toxicity, and high mortality. Ultrasound imaging is a rapid and non-invasive tool that can be used to help make the diagnosis of necrotizing fasciitis by identifying several distinctive sonographic findings. The purpose of this study is to describe the construction of a realistic diagnostic training model for necrotizing fasciitis using fresh frozen cadavers and common, affordable materials.

Presently, fresh non-embalmed cadavers have been used at medical institutions for various educational sessions including cadaver-based ultrasound training sessions. Details for the preparation and construction of a necrotizing fasciitis cadaver model are presented here. This paper shows that the images obtained from the cadaver model closely imitate the ultrasound appearance of fluid and gas seen in actual clinical cases of necrotizing fasciitis.

Therefore, it can be concluded that this cadaver-based model produces high-quality sonographic images that simulate those found in true cases of necrotizing fasciitis and is ideal for demonstrating the sonographic findings of necrotizing fasciitis.

## Introduction

Ultrasound is a rapid and non-invasive tool ideal for the imaging of soft tissue infections, and it has been associated with a change in clinician management in 50% of emergency department soft tissue infections [1]. Necrotizing fasciitis (NF) is a devastating infectious disease process characterized by the rapid spread of inflammation, extensive soft tissue necrosis along deep fascial planes, systemic toxicity, and high mortality. Classic physical exam findings of NF include edema, erythema, warmth, blistering, and crepitus; however, the early stages of infection have little overlying skin changes [2]. Early identification and treatment of NF has been shown to decrease morbidity and mortality; as a result, it is important for practitioners to rapidly identify and accurately diagnose NF. Ultrasound is a diagnostic tool that has been used to help clinicians with early identification of NF. NF has several distinctive sonographic manifestations, and the identification of these findings can aid in an expedited diagnosis.

Although various diagnostic imaging modalities such as computed tomography (CT) and magnetic resonance imaging (MRI) are often used in the diagnosis of necrotizing fasciitis (NF); the diagnostic gold standard for NF relies on surgical exploration and tissue cultures (the most common organisms are Streptococcus, Staphylococcus aureus, and Enterococcus species) [3]. Due to the rarity of this highly morbid disease, simulation models have been devised to improve the critical thinking and diagnostic acumen of students and physicians [4]. Criticisms of these synthetic or simulation models point out that they have less realistic tactile features and do not integrate with bedside imaging which is often used for soft tissue infections. Currently, there are no realistic models that provide the learner with the opportunity to evaluate and identify the sonographic findings for NF. With the increased utilization of diagnostic ultrasound in medical practice and residency training, educational models must incorporate the use of ultrasound in these medical training models [5-10]. Teaching models for highly morbid and rare conditions such as NF have been shown to improve diagnostic accuracy as well as clinical management of such patients, and in particular, cadaver-based models have been shown to improve ultrasound-guided techniques [5,10]. The objective of this study is to describe the construction of a cadaver-based model designed to aid student and clinician learning of the sonographic features associated NF: soft tissue thickening, fluid collections along the fascial planes, and gas in the deep soft tissues [11-12]. Details for the construction of the diagnostic training model for NF using common and affordable materials are presented below.

## Technical report

Fresh non-embalmed cadavers are used at medical institutions, including ours, for various educational sessions including cadaver-based ultrasound training sessions [5-10].

### Materials

The following materials are needed to prepare this training model: A fresh cadaver, ultrasound gel, normal saline, and an 18 guage needle with a 20 cc (or larger) syringe.

### Model

**Figure 1:**
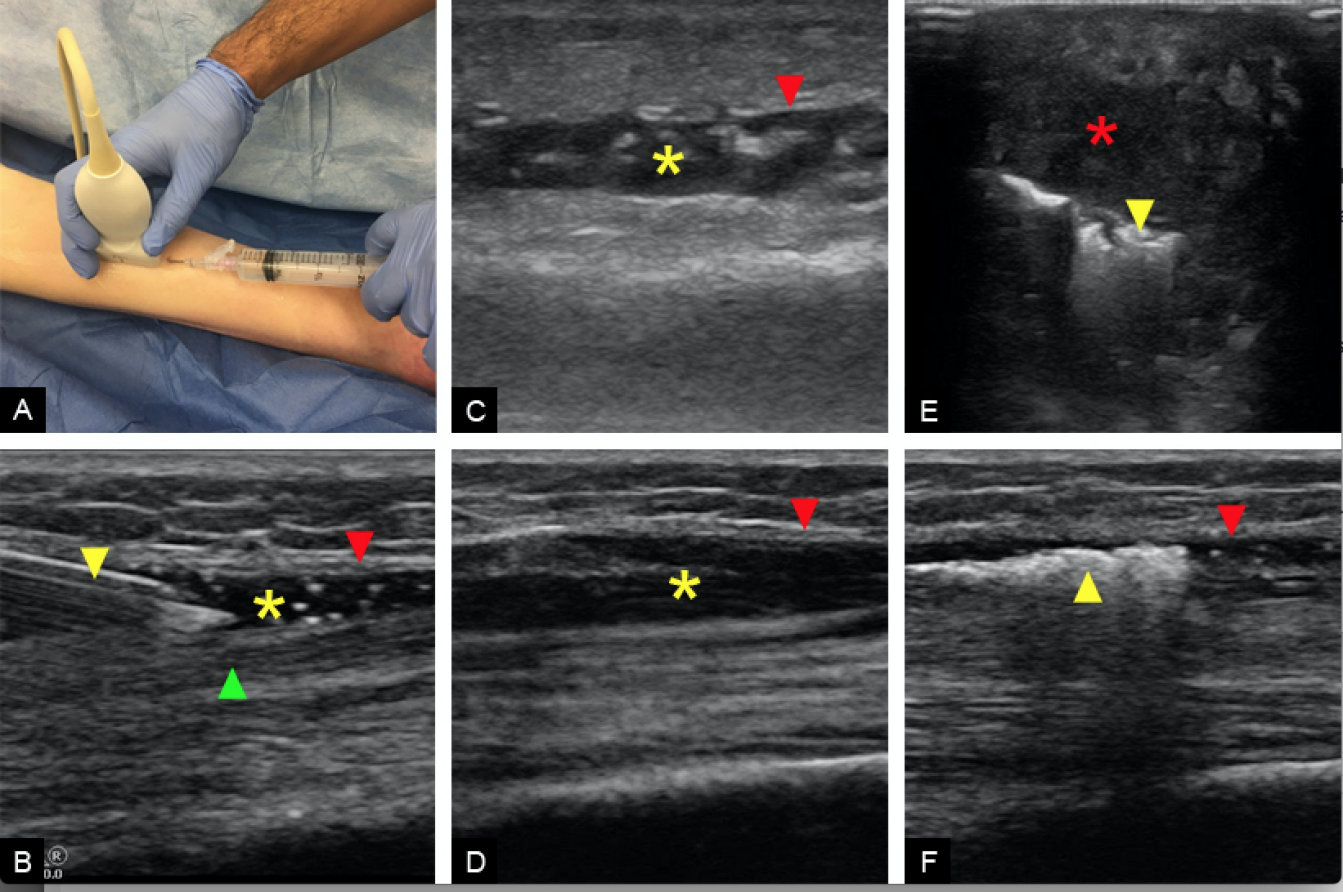
Preparation and Results A) Setup of the cadaver model showing ultrasound guided needle entry into the desired fascial plane. B) Sagittal ultrasound of the anterior lower extremity of the cadaver model showing needle guidance into the crural fascia; 18 gauge needle (yellow arrowhead), crural fascia (red arrowhead), tibialis anterior (green arrowhead), 1:1 mixture within fascia (yellow asterisk). C) Sagittal ultrasound of a real case of NF in the lower extremity showing fluid collection (yellow asterisk) below the crural fascia (red arrowhead). D) Cadaver model sagittal ultrasound of the lower extremity showing fluid collection (yellow asterisk) below the crural fascia (red arrowhead). E) Transverse ultrasound of a real case of NF in the foot showing hyperechoic gas collection (yellow arrowhead) within the plantar fascia deep to the subcutaneous tissue (red asterisk). F) Cadaver model sagittal ultrasound of the lower extremity showing hyperechoic gas collection (yellow arrowhead) within the crural fascia (red arrowhead).

To create the NF model, the following steps are taken: 1) Ultrasound the desired musculoskeletal region of interest (Figure 1A) and identify the fascial planes. 2) Under direct sonographic visualization, guide the 18 gauge needle into the fascial plane and inject a 1:1 mixture of ultrasound gel and saline (Figure 1B). Be sure to separate the fascial planes and negotiate the needle as necessary. It is important to inject within fascial planes as opposed to within a muscle belly. Inject a sufficient amount of solution so that the fascial plane has > 7mm of free fluid. (Facial planes in the forearm may require 10-20 cc of fluid whereas thigh fascia may require 30-50 cc of fluid.) In the event of incidental intramuscular injection, advance or withdraw the needle in order to successfully separate facial planes. In the event of a failed injection, ultrasound a new anatomical section and evaluate a new region and repeat. In the authors' experience, success is 100%. 3) Once the facial planes are separated by fluid, inject air into the fluid-filled fascial space. (5-10 cc of air is sufficient but may require additional injections.)

### Results

**Figure 2:**
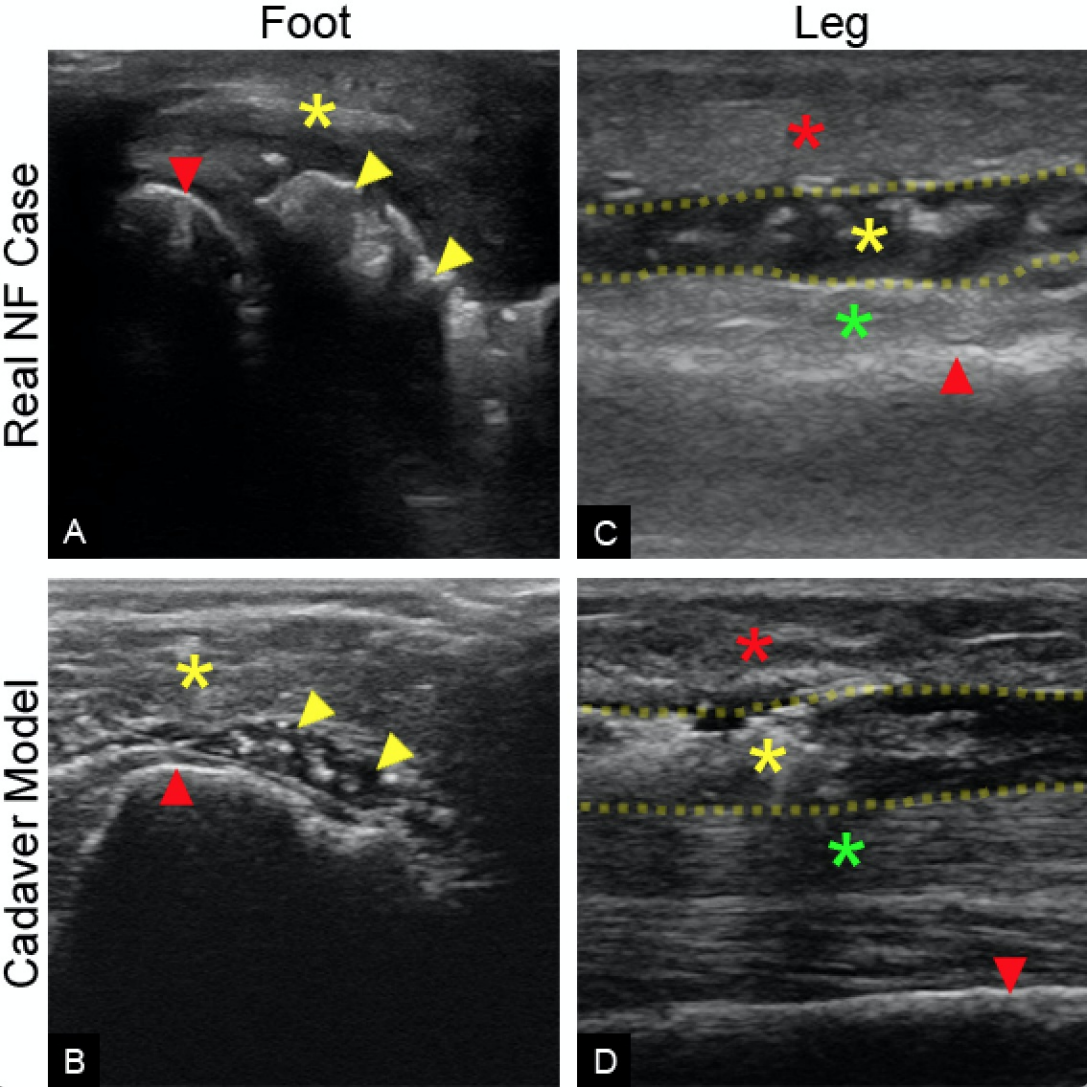
Sonographic Comparisons Ultrasound comparison of the cadaver model and real cases of NF in the foot and leg showing a very similar sonographic appearance. A) Long-axis ultrasound of a real case of NF in the foot showing gas collection (yellow arrowheads) distal to the calcaneus (red arrowhead) and deep to the subcutaneous tissue (yellow asterisk). B) Long-axis ultrasound of the cadaver model of the foot showing gas and fluid collection (yellow arrowheads) distal to the calcaneus (red arrowhead) and deep to the subcutaneous tissue (yellow asterisk). C) Sagittal ultrasound of a real case of NF in the leg showing subcutaneous tissue (red asterisk), fluid collection (yellow asterisk) within crural fascia (yellow dotted line), tibialis anterior (green asterisk), and tibia (red arrowhead). D) Sagittal ultrasound of the cadaver model of the leg showing subcutaneous tissue (red asterisk), fluid collection (yellow asterisk) within crural fascia (yellow dotted line), tibialis anterior (green asterisk), and tibia (red arrowhead).

The surface quality of a fresh cadaver is certainly realistic; as such, the model mimicked the tactile feel of a true clinical scenario quite well. Furthermore, the unique anatomy of each cadaver was similar to the variable anatomy found in true clinical encounters. The cadaver skin lacked warmth and erythema; however, this was determined to be beneficial given that many cases of NF do not present with these symptoms. The cadaver model closely mimicked the ultrasound appearance of fluid and gas as seen in actual clinical cases of NF (Figure 1C-1F). The desired location for NF was well maintained even with repeated palpation and sonographic examination. The NF simulation was successfully placed in several anatomic locations as well as at varying fascial depths thereby simulating various clinical scenarios and appearances (Figure 2).

## Discussion

Necrotizing fasciitis is a devastating soft tissue infection characterized by the rapid spread of inflammation and necrosis that progressively involves the deep fascia, subcutaneous fat, overlying skin, and even skeletal muscle [2-3]. Because of the mostly avascular nature of fascia, bacteria can rapidly propagate throughout the body using the fascia as a makeshift highway system. Although often associated with the notorious Group A Streptococci (the so-called “flesh-eating bacteria”), 55%-75% of all cases are caused by a polymicrobial anaerobic infection [13]. NF is fortunately rare in comparison to other soft tissue infections such as cellulitis, with an incidence of approximately 500-800 cases per year in the United States [4]. However, unlike more superficial soft tissue infections, NF is limb-threatening and can lead to systemic toxicity, multi-organ failure, and death [2-3,14]. Although the case fatality rate has decreased with modern advances in critical care medicine, it still remains as high as 76% [2-3,15]. Prompt diagnosis of NF can be difficult since early in its course it can appear deceptively benign. The early stages may only reveal subtle findings (localized pain, swelling, tenderness, and warmth) which may make NF difficult to distinguish from other superficial soft tissue infections (such as cellulitis or erysipelas). NF can present simply with pain out of proportion to physical exam findings (the most consistent initial presenting symptom), but other presenting symptoms include swollen erythematous skin with poorly demarcated borders and fever. Many of the classic physical exam findings of NF such as blistering, skin crepitus, visible necrosis, and gas bullae are late findings [2-3]. Common comorbidities include diabetes mellitus, immunosuppression, intravenous drug use, skin injury, peripheral vascular disease, and end-stage renal, pulmonary, and liver disease [3,11,14].

The diagnostic gold standard for NF is surgical exploration with tissue cultures, and its treatment centers around source control through aggressive necrotic tissue debridement, fluid maintenance, and appropriate antibiotic therapy [2-3]. While the laboratory risk indicator for necrotizing fasciitis (LRINEC) score has been shown to be highly specific with a high negative predictive value, newer studies have shown that it is not adequately sensitive [16-17]. Plain radiographs may show thickening of soft tissues and sometimes soft-tissue gas, but they rarely contribute to the diagnosis [18]. Contrast-enhanced CT scans may be comparable to MRIs, but their utility is limited by the common concurrence of acute kidney injury in this patient population [4,18-19]. MRI is historically the preferred diagnostic imaging modality for NF [4,18,20]. The absence of deep fascial involvement on MRI can exclude NF (100% sensitive, 61% specific), and the presence of gas is diagnostic (100% specific, 43% sensitive). However, MRIs tend to overestimate deep fascial involvement and have poor specificity; additionally, it is costly, time-consuming, and not always readily available [12,18,20]. Therefore, relying on MRIs for the diagnosis can pose significant and potentially lethal delays in patient care management plans [12]. Early diagnosis of NF is ultimately the most important factor affecting outcomes which can be problematic given the subtle initial presentation [2-3]. Surgery should never be delayed in favor of additional imaging and laboratory testing if clinical suspicion is sufficiently high [14].

Point of care ultrasound has proven to be useful in rapid clinical decision-making scenarios and has gained popularity because it is fast, non-radiating, and readily available [11]. Studies show it to be highly sensitive and specific (88% sensitive and 93% specific) for NF based on certain imaging criteria, such as soft tissue thickening, fascial fluid accumulation greater than 4 mm, and the presence of subcutaneous air [12]. In some cases involving non-gas producing Group A Streptococci infections, ultrasound has been shown to help diagnose NF with negative findings on CT and MRI [6]. Given that NF is rare, difficult to diagnose, and associated with increased mortality with delayed diagnosis, it is imperative that clinicians take immediate action such as promptly initiating surgical consultation when suspecting NF [14]. Point of care ultrasound is therefore an additional tool that can help expedite the diagnosis and provide information without delaying patient care [11].

Since ultrasound imaging can be highly operator dependent, suitable teaching models should be available to properly train clinicians on how to best diagnose and recognize sonographic findings of NF. A mannequin-based model for NF (in addition to plain radiographs and CT scans demonstrating NF) has been shown to improve diagnostic ability of learners; however, this study did not include the use of ultrasound [4]. To our knowledge, the model described in this manuscript is the first cadaver-based simulation tool to help clinicians learn to diagnose necrotizing fasciitis using bedside ultrasound. The anatomically realistic cadaveric model described in this paper is ideal to expose students and clinicians to NF in a non-urgent situation so that they may be better adapted to diagnose this rare yet dangerous disease process.         

### Limitations

The major limitation of this model is the cost and availability of cadavers since we are dependent upon the Willed Body Program and the monumental and generous gifts of our donors. As with most cadaver-based educational sessions, it is prudent to maximize the number of training simulations. We did not evaluate the effectiveness of the model for teaching and retaining the use of ultrasound as a diagnostic tool for NF, although these issues will be topics for further investigation using our cadaver model.

## Conclusions

Necrotizing fasciitis is a disease with significant risk for morbidity and mortality. With the ubiquitous use of ultrasound in emergency departments, it is pivotal that emergency clinicians be aware of the sonographic appearance of NF on ultrasound. Our model successfully replicates the clinical and ultrasound appearance of necrotizing fasciitis as compared to real clinical cases. Our model is well suited to simulate how point of care ultrasound can be used to aid in the diagnostic workup of necrotizing fasciitis while minimizing time to surgical consultation and intervention.

## References

[1] Tayal VS, Hasan N, Norton HJ, Tomaszewski CA (2006). The effect of soft-tissue ultrasound on the management of cellulitis in the emergency department.. Acad Emerg Med.

[2] Sarani B, Strong M, Pascual J, Schwab CW (2009). Necrotizing fasciitis: current concepts and review of the literature. J Am Coll Surg.

[3] Lancerotto L, Tocco I, Salmaso R, Vindigni V, Bassetto F (2012). Necrotizing fasciitis: classification, diagnosis, and management. J Trauma Acute Care Surg.

[4] Wall DB, Klein SR, Black S, De Virgilio C (2000). A simple model to help distinguish necrotizing fasciitis from nonnecrotizing soft tissue infection. J Am Coll Surg.

[5] Hoyer R, Means R, Robertson J, Rappaport D, Schmier C, Jones T, Stolz LA, Kaplan SJ, Adamas-Rappaport WJ, Amini R (2016). Ultrasound-guided procedures in medical education: a fresh look at cadavers. Intern Emerg Med.

[6] Amini R, Adhikari S, Fiorello A (2014). Ultrasound competency assessment in emergency medicine residency programs. Acad Emerg Med.

[7] Amini R, Stolz LA, Kartchner JZ, Thompson M, Stea N, Hawbaker N, Joshi R, Adhikari S (2016). Bedside echo for chest pain: an algorithm for education and assessment. Adv Med Educ Pract.

[8] Amini R, Stolz LA, Javedani PP, Gaskin K, Baker N, Ng V, Adhikari S (2016). Point-of-care echocardiography in simulation-based education and assessment. Adv Med Educ Pract.

[9] Amini R, Stolz LA, Hernandez NC, Gaskin K, Baker N, Sanders AB, Adhikari S (2016). Sonography and hypotension: a change to critical problem solving in undergraduate medical education. Adv Med Educ Pract. 2016 Jan 1.

[10] Miller R, Ho H, Ng V, Tran M, Rappaport D, Rappaport WJ, Dandorf SJ, Dunleavy J, Viscusi R, Amini R (2016). Introduction of a fresh cadaver model for ultrasound-guided central venous access for training during the third year of medical school. West J Emerg Med.

[11] Kehrl Kehrl, T T (2014). Point-of-care ultrasound diagnosis of necrotizing fasciitis missed by computed tomography and magnetic resonance imaging. J Emerg Med.

[12] Yen ZS, Wang HP, Ma HM, Chen SC, Chen WJ (2002). Ultrasonographic screening of clinically-suspected necrotizing fasciitis. Acad Emerg Med.

[13] Dixon B (2008). Fasciitis continues to surprise. Lancet Infect Dis.

[14] Wong CH, Chang HC, Pasupathy S, Khin LW, Tan JL, Low CO (2003). Necrotizing fasciitis: clinical presentation, microbiology, and determinants of mortality. J Bone Joint Surg Am.

[15] Dworkin M, Westercamp M, Park L, McIntyre A (2009). The epidemiology of necrotizing fasciitis including factors associated with death and amputation. Epidemiol Infect.

[16] Holland MJ (2009). Application of the laboratory risk indicator in necrotising fasciitis (LRINEC) score to patients in a tropical tertiary referral centre. Anaesth Intensive Care.

[17] Burner E, Henderson SO, Burke G, Nakashioya J, Hoffman JR (2016). Inadequate sensitivity of the laboratory risk indicator to rule out necrotizing fasciitis in the emergency department. West J Emerg Med.

[18] Malghem J, Lecouvet FE, Omoumi P, Maldague BE, Vande Berg BC (2013). Necrotizing fasciitis: contribution and limitations of diagnostic imaging. Joint Bone Spine.

[19] Sharif H, Clark D, Aabed M, Aideyan O, Haddad M, Mattsson T (1990). MR imaging of thoracic and abdominal wall infections: comparison with other imaging procedures. AJR Am J Roentgenol.

[20] Schmid MR, Kossmann T, Duewell S (1998). Differentiation of necrotizing fasciitis and cellulitis using MR imaging. AJR Am J Roentgenol.

